# A blinded, randomized, placebo-controlled trial of the safety of oclacitinib in cats

**DOI:** 10.1186/s12917-019-1893-x

**Published:** 2019-05-08

**Authors:** Natália Lôres Lopes, Diefrey Ribeiro Campos, Marília Alves Machado, Mariana Silva Revoredo Alves, Manuela Silva Gomes de Souza, Cristiano Chaves Pessoa da Veiga, Alexandre Merlo, Fábio Barbour Scott, Julio Israel Fernandes

**Affiliations:** 1 Veterinary Medicine, Universidade Federal Rural do Rio de Janeiro (UFRRJ), Seropédica, Brazil; 2 Veterinary Science, UFRRJ, Seropédica, Brazil; 3 School of Veterinary Medicine, UFRRJ, Seropédica, Brazil; 4 Veterinary Hospital, UFRRJ, Seropédica, Brazil; 5 Zoetis Industry of Veterinary Products, São Paulo, Brazil; 6 Animal Parasitology Department, UFRRJ, Seropédica, Brazil; 7 Veterinary Medicine and Surgery Department, UFRRJ, Seropédica, Brazil

**Keywords:** Cats, Clinical effects, Feline, Oclacitinib, Pruritus, Safety

## Abstract

**Background:**

Oclacitinib is a Janus kinase (JAK) 1 enzyme inhibitor and blocks JAK1-dependent cytokines and is used to control pruritus. Studies available in cats are very limited and as there is a potential role for oclacitinib in the control of pruritus in this specie, the aim of this study was to evaluate the safety and clinical effects of oral oclacitinib maleate in healthy cats.

**Results:**

Thirty mixed-breed cats weighing from 2.1 to 5.3 kg each were randomly allocated to three treatment groups of 10 animals each. Cats in two groups received oclacitinib at 1 mg/kg or 2 mg/kg q 12 h orally for 28 days. Cats in the third group were given placebo tablets (cornstarch) q 12 h orally for 28 days. Oclacitinib maleate was well tolerated during the study and few adverse events were observed in treated cats. Clinical signs of toxicity were not observed in any animals treated at 1 mg/kg. Gastrointestinal clinical signs observed in the 2 mg/kg group included vomiting in two of the 10 cats and soft stools in two cats. One cat treated with placebo also exhibited soft stools. No significant differences were observed between the groups for hematologic analyses performed during the study. There was a slight increase in neutrophils and monocytes and a decrease in eosinophil mean counts in treated cats. Mean renal and liver enzymes remained normal throughout the entire study. A small, but significant increase in fructosamine levels was observed for both treated groups compared with placebo; however, values remained within the normal reference range. There were no significant difference between treated groups and the placebo group for urine specific gravity, pH, or urine protein to creatinine ratio mean values.

**Conclusions:**

Oclacitinib maleate was well tolerated by cats at 1 mg/kg and 2 mg/kg and appeared to be safe for this species when administered orally twice daily for 28 days. More studies would be needed to demonstrate if oclacitinib maleate may be a suitable alternative to treat pruritic cats.

## Background

Pruritus, the most common clinical sign observed in dermatology [1], is present in hypersensitivity diseases such as allergic dermatitis. In cats, these disorders are classified as flea bite hypersensitivity, food-induced hypersensitivity dermatitis and “non-flea, non-food” hypersensitivity dermatitis (NFNFHD) [2].

Treatment options for allergic dermatitis include glucocorticoids, cyclosporine, antihistamines, essential fatty acids, and immunotherapy [1, 3]. Oral (prednisolone) and injectable glucocorticoids are available to control pruritus in cats; however, side effects, such as diabetes mellitus, Cushing’s disease, skin fragility, and diarrhea may be observed [3]. Methylprednisolone and triamcinolone were reported effective and well tolerated glucocorticoids, but side effects like a significant increase in serum fructosamine and in serum albumin were observed [4]. It is recommended that an immediate reduction in the dosage of glucocorticoids is made. Cats in long-term treatment should be examined regularly, and urinalysis and blood chemistry testing should be performed [5].

Cyclosporine also is shown to be effective in cats [6–8], but initial improvement may only occur during the second week of treatment [5], with 4 to 8 weeks needed to achieve a satisfactory clinical response [3]. Side effects, such as vomiting, diarrhea, loose stools, and hypersalivation related to oral cyclosporine administration were reported by some authors [6, 7]. Recently, a subcutaneous administration of cyclosporine in allergic cats was effective and suggested as an alternative to oral administration, but the development of lesions associated with injections sites were observed as adverse effects [8]. This drug is also associated with the development of *Toxoplasma gondii* infection [9, 10].

Oclacitinib is a Janus kinase (JAK) 1 enzyme inhibitor and blocks JAK1-dependent cytokines, such as IL-2, IL-4, IL-6, IL-13, and IL-31 involved in allergy, inflammation, and pruritus [11]. In a canine IL-31 pruritus model, anti-pruritic activity of this drug was greater than that of both prednisolone and dexamethasone [12]. Oclacitinib is shown to be effective in the treatment of canine atopic dermatitis [13–15]. Results of earlier studies demonstrated a rapid antipruritic effect by oclacitinib, with a reduction of pruritus within 24 h [13], a faster onset of action than that of cyclosporine [16].

Oclacitinib is well tolerated by dogs, and adverse effects included vomiting and diarrhea [14], but with a lower frequency than those observed with administration of cyclosporine [16]. Long-term administration was shown to be safe and effective, with an outcome of improved the quality of life of dogs [15].

Much less is known about feline allergic skin disease [2]. However, in an experimental model using IL-31–induced pruritus in cats oclacitinib given at 0.4 mg/kg or 1 mg/kg 1 h before administration of this interleukin reduced pruritus in 63 and 62% of the test animals, respectively [17]. In the treatment of NFNFHD, oclacitinib administered at 0.4 to 0.6 mg/kg may suppress pruritus and clinical signs related to allergic dermatitis; however, it has been suggested that a higher dose or a different dosing regimen may improve the response [18]. A higher dose of 1 mg/kg given twice daily for 31 days was reported to provide a good clinical response in a case of feline cutaneous mastocytosis with no adverse effects observed [19]. In cats with experimental asthma, oclacitinib at 0.5 mg/kg or 1 mg/kg twice daily for 28 days significantly suppressed airway inflammation and adverse clinical signs were not observed [20]. Oclacitinib also was successfully used in a case report of feline idiopathic ulcerative dermatitis at dosages of 1.5–2 mg/kg/day [21]. Dosages ranging from 0.8–1.3 mg/kg twice daily were effective in cats with NFNFHD [22].

As there is a potential role for oclacitinib in the control of pruritus in cats, and studies available in this specie are very limited, the aim of this study was to evaluate the safety and clinical effects of this drug in healthy cats. This was the first blinded, randomized, placebo-controlled trial to evaluate the safety of oclacitinib in healthy cats to be published.

## Results

Mean dosages ± standard deviation for treated oclacitinib groups were 1.02 mg ± 0.104 mg for 1 mg/kg q 12 h group and 2.002 mg ± 0.076) for 2 mg/kg q 12 h group.

### Clinical signs

Primary clinical data are summarized in Table 1. Cats in the 1 mg/kg group presented no clinical signs during the study. Vomiting occurred in two animals from 2 mg/kg group, one of which occurred only on one day; the second cat vomited on several days of observation. Transient soft stools were observed in two cats from 2 mg/kg group and one from placebo group. Fecal samples collected and analyzed from those two cats in 2 mg/kg group were positive for *Giardia* sp. A cat from placebo group developed an erythematous cutaneous lesion on day 7. All cats had normal urination, normal appetite, and food consumption throughout the study, through daily examination and characterized as normal or abnormal.

**Table 1:** Clinical findings observed in cats in treated with oral oclacitinib or placebo

Clinical findings	Placebo	1 mg/kg	2 mg/kg
Vomiting	0/10	0/10	2/10
Soft stools	1/10	0/10	2/10
Increased lymph node size	1/10	1/10	1/10
Cutaneous lesion	1/10	0/10	0/10

Data are expressed as the number of cats with the clinical sign over total cats in the group

Mean body weights increased in all three groups throughout the study, ranging from 0.21 kg in the 1 mg/kg group to 0.29 kg in cats treated with placebo with no significant differences between the groups. The average respiratory rate was above the reference range; however, there were no significant differences between the groups. One cat of each group presented pale mucosa, which was observed on only one occasion for each cat. Increased popliteal lymph node size was observed by palpation occurred in three cats, including one in the 1 mg/kg group (one day), one cat in the 2 mg/kg group (for two days) and one cat from the placebo group. Axillary lymph nodes were increased in size for one animal from placebo group on day 7. Stomatitis was observed in one cat from placebo group on day 42 (14 days post treatment). All the other clinical parameters remained within the reference range, with no significant differences between groups.

### Hematology, serum chemistry, and urinalysis

Hematology data for selected sampling days are displayed in Table 2. Overall, deviations from normal reference ranges or findings of significant differences between groups treated with oclacitinib and the placebo group were minor and transient, with no trends apparent for any parameters evaluated in the hematologic profile. Mean values for red blood cell count, hemoglobin, and hematocrit were within the normal reference range for all three groups. Mean platelet counts decreased below the reference range on day 42 (mean 128,500/mm^3^) in the 1 mg/kg group and on days 3 (mean 118,000/ mm^3^) and 42 (mean 124,200/ mm^3^) in the placebo group; however, there were no significant differences between groups at any sampling time.

**Table 2:** Hematology mean values observed in cats treated with oral oclacitinib or placebo

Parameter	Placebo					1 mg/kg					2 mg/kg				
	Day −2	Day 7	Day 14	Day 28	Day 42	Day −2	Day 7	Day 14	Day 28	Day 42	Day −2	Day 7	Day 14	Day 28	Day 42
RBC (× 10^6^ /μL)	9.53	9.05	9.71	9.34	9.75	9.06	8.62	9.19	9.16	9.25	9.39	9.69	9.39	9.02	9.63
Hemoglobin (g/dL)	11.81	11.64	12.55	11.66	12.10	12.18	11.78	12.27	11.66	11.94	12.38	12.75	11.97	11.60	12.40
Hematocrit (%)	39.13	36.35	40.49	38.76	39.11	38.97	37.54	39.03	39.63	38.10	40.00	39.38	40.00	38.12	39.67
WBC (/μL)	10,887	12,564	12,640	12,620	12,090	12,410	15,760	14,910	14,970	14,070	11,160	13,430	13,330	11,460	11,010
Neutrophils	6232.5	7590	5608.6	6983.7	6390.0	7662.3	8595.5	8877.4	7804.9	9168.9	6071.6	7179.4	6823.5	5635.7	6235.8
Lymphocytes	3556.8	4615.1	5049.0	4619.1	4609.6	3546.6	5924.3	4830.4	4968.5	3737.4	3753.0	6094.1	5334.6	5002.4	3468.3
Monocytes	208.6	301.0	448.2	329.7	237.0	205.8	280.2	293.3	361.6	353.2	199.4	287.1	222.5	209.4	261.7
Eosinophils	695.2	825.70	850.2	1040.5	831.2	1257.3	960.0	908.9^a^	962.4	810.5	1136.2	689.4	470.1^a^	612.5	1033.3
Platelets (/μL)	161,900	186,100	165,600	170,100	124,200	162,900	170,000	162,900	161,200	128,500	181,300	190,800	155,300	265,700	162,200

*RBC* red blood cells, *WBC* white blood cells

^a^Significantly different than placebo value at the same day (*p* = 0.04)

Reference values: RBC-6.0-10.0; hemoglobin- 9.5-15.0; hematocrit- 29-45; WBC- 5.5-19.5 × 10^3^; neutrophils-2500-12,500; lymphocytes- 1500-7000; monocytes- 0-850; eosinophils-0-1500; platelets- 150-600 × 10^3^

Mean white blood cell counts were within the reference range, with the exception of an increase on day 21 in all groups. There were no significant differences in white blood cell counts between groups at any sampling time. Neutrophil mean values also were within the reference range, but there was a transient significant increase on day 21 (*p* = 0.007) for both treated groups compared with the placebo group. Also on day 21, mean monocyte counts were significantly higher in 1 mg/kg group than in the placebo group (*p* = 0.03) and significantly lower in 2 mg/kg (p = 0.03); however, values were within the normal reference range. The mean lymphocyte count was within the normal reference range at all samplings for cats in all groups, with no significant differences between placebo and treated cats. A decrease in the mean eosinophil count was observed in both oclacitinib groups, and this decrease was significant between the placebo and treated groups on day 14 (*p* = 0.04), but the mean value remained within the normal reference range for all days.

Mean serum chemistry results for selected sampling days are shown in Table 3. As with hematologic findings, deviations from normal reference ranges or occurrence of significant differences between groups treated with oclacitinib and the placebo group were minor and transient, with no trends apparent for any parameters evaluated in the serum chemistry profile. In 1 mg/kg group, alanine aminotransferase (days 7 and 28), total bilirubin (days 7 and 14), and direct bilirubin (day 7) were above the reference range. Alanine aminotransferase and direct bilirubin were higher than for the placebo group on day 14 and indirect bilirubin was higher than the placebo group day 7. Triglyceride levels were slightly above reference range in 1 mg/kg group on day 7 and in placebo group on day 14. Cholesterol, albumin, total protein, glucose, urea, and creatinine mean levels were normal in all groups throughout the study. There was no significant difference in mean clinical chemistry parameters between the groups, except for fructosamine on day 7 (*p* = 0.03), with a slight increase in both treated groups compared with placebo, but within the normal reference range.

**Table 3:** Serum chemistry mean values observed in cats treated with oral oclacitinib or placebo

Parameter	Placebo					1 mg/kg					2 mg/kg				
	Day −2	Day 7	Day 14	Day 28	Day 42	Day −2	Day 7	Day 14	Day 28	Day 42	Day − 2	Day 7	Day 14	Day 28	Day 42
Alkaline phosphatase (U/l)	33.0	30.2	27.0	27.8	35.1	28.4	25.7	27.5	32.7	32.7	33.7	34.2	33.6	32.3	36.4
ALT (U/l)	71.2	76.2	87.4	65.5	61.9	75.1	85.5	75.0	76.9	55.7	49.4	54.0	56.0	63.5	60.7
AST (U/l)	27.6	30.2	29.4	24.2	27.7	31.5	28.9	34.0	32.5	30.7	26.3	31.6	27.7	28.0	34.8
Total bilirrubin (mg/dL)	0.59	1.14	1.21	0.49	0.53	0.56	1.11	0.84	0.42	0.52	0.58	0.50	0.79	0.61	0.55
Direct bilirrubin (mg/dL)	0.32	0.34	0.55	0.23	0.21	0.24	0.54	0.37	0.19	0.20	0.25	0.25	0.36	0.27	0.22
Indirect bilirrubin (mg/dL)	0.27	0.80	0.66	0.26	0.32	0.32	0.57	0.47	0.23	0.32	0.33	0.26	0.43	0.34	0.33
GGT (U/l)	0.0	0.1	0.0	0.0	0.1	0.0	0.0	0.2	0.0	0.0	0.2	0.1	0.0	0.1	0.0
Glucose (mg/dL)	75.6	76.4	76.0	71.5	77.3	86.0	74.9	74.6	72.2	78.0	87.5	8.7	78.0	72.0	80.9
Fructosamine (μ mol/l)	244.80	246.63	229.32	252.58	249.90	267.15	267.33^a^	262.98	263.55	269.82	236.57	252.45^a^	240.37	238.50	261.95
Triglycerides (mg/dL)	58.6	65.8	94.0	47.0	62.8	56.2	96.8	76.3	44.4	49.7	54.7	45.2	71.2	51.2	66.8
Cholesterol (mg/dL)	105.4	11.1	116.3	102.9	108.8	102.7	111.9	116.2	101.8	107.3	106.9	121.8	131.6	112.9	111.5
Albumin (g/dL)	2.24	2.24	2.40	2.24	2.45	2.25	2.27	2.36	2.15	2.38	2.17	2.26	2.35	2.27	2.40
Total protein (g/dL)	6.44	6.54	6.86	6.58	6.87	6.70	6.83	6.93	6.23	6.76	6.38	6.51	6.50	6.31	6.63
Urea (mg/dL)	43.5	44.1	51.1	43.3	42.9	43.6	43.4	48.5	42.8	41.7	42.8	48.40	50.2	45.8	41.5
Creatinine (mg/dL)	1.25	1.23	1.29	1.36	1.24	1.42	1.30	1.39	1.39	1.34	1.35	1.41	1.36	1.42	1.39

ALT, alanine aminotransferase; AST, aspartate aminotransferase; GGT, gamma glutamyl transpeptidase

^a^Significantly different than placebo value at the same day (*p* = 0.03)

Reference values: alkaline phosphatase-0-62; ALT-28-76; AST-5-55; total bilirubin-0.1-0.4; direct bilirubin-0.04-0.3; indirect bilirubin-0.01-0.5; GGT-1-7; glucose-70-150; fructosamine- 190-400; triglycerides-20-90; cholesterol-82-218; albumin-2.4-4.1; total protein-5.9-8.5; urea-42.8-64.2; creatinine-0.8-1.6

There was no significant difference for the treated groups compared with the placebo group for urine specific gravity, pH, or urine protein to creatinine ratio (UP/C) mean values. Hyaline casts were observed in two cats of the 1 mg/kg group on day 28), in two cats of the 2 mg/kg group on days 28 and 42 and in one cat on day 42, and in one cat from the placebo group on day − 2 and one cat on day 28.

Granular casts occurred in two cats in the 2 mg/kg group (one on days − 2 and 14 and one on day 42) and in two cats in the placebo group (one on day 28 and one on day 42). Fatty casts were noted in one cat in the 1 mg/kg group on day 42 and in one cat in the 2 mg/kg group on day 28. All casts were present in low numbers (rare). Struvite crystals were present in three cats from the 1 mg/kg group (one cat each on days 14, 28, and 42), two cats in the 2 mg/kg group (one cat on day 28 and one on days 28 and 42), and three cats in the placebo group (two on day 14, one on 28 and one on 42). Calcium oxalate crystals were observed in one cat from 2 mg/kg group on day 28 and one from placebo on day − 2. Glycosuria was not observed in any cats during the study.

### Ultrasound

Abdominal ultrasound showed no alteration in the stomach, intestines, or liver in any cats. Only one cat from 1 mg/kg group exhibited higher echogenicity for both kidneys on days 28 and 42. Cellularity was observed in the ultrasound of the bladder in 7 cats from each group.

## Discussion

The dosages of oclacitinib of 1 mg/kg and 2 mg/kg were chosen because the dosages used for dogs (0.4–0.6 mg/kg) were not shown to be very effective in cats [18], and the higher dosage range of 0.8–1.3 appeared to be more effective in allergic cats [22]. There are no published studies of pharmacokinetics study of this drug in cats, and more investigations are needed to evaluate whether there is a possibility of cats having a longer half-life or less sensitivity to oclacitinib.

In this study, oclacitinib was well tolerated and with very few adverse events. Those that were recorded were minor and transient in nature. Vomiting was observed only with the higher dose (2 mg/kg) and in only two animals, one of which vomited only once. This adverse effect was previously reported in dogs with allergic skin disease receiving treatment with oral oclacitinib as well [13–15]. This observation was no longer evident at the end of the treatment period.

Diarrhea was previously reported to occur in dogs treated with oclacitinib [13–15]. Soft stools occurred in two cats from the 2 mg/kg group but was also observed in one cat from placebo group. This finding was isolated and resolved spontaneously with no need to interrupt the medication. Another factor to consider is that fecal samples obtained from those treated cats were positive for *Giardia* on the days soft stools were observed, and this parasite is commonly associated with the development of diarrhea [23]. Fecal samples were not collected for testing before initiation of the trial, leading to the possibility that the cats may have had the infection before the study. It is noteworthy the cats remained confined throughout the study and eventually stress may have triggered the release of cysts, in the otherwise asymptomatic animals [24]. Therefore, it is not possible to attribute this clinical sign with certainty to treatment with oclacitinib. The cats were treated for *Giardia* after the study ended.

Increased lymph node size observed in cats from both treated groups was transient and resolved spontaneously. Increased lymph node size also was observed from a cat in the placebo group. After the end of the study, the lymph nodes returned to the normal size. Therefore, this finding could not be definitely attributed to treatment with oclacitinib or clinical correlation and was attributed to a nonspecific clinical finding. The high respiratory rate observed every day of observations and sampling in all three groups could be attributed to excitement and anxiety during the procedures. The weight gain in cats of all three groups was likely due to cage confinement during the trial and unrelated to oclacitinib administration. Clinical signs such as pyoderma, yeast infection, and otitis reported in atopic dogs that received oral oclacitinib, were probably related to the atopic dermatitis itself [13]. and were not observed in the treated cats during the present trial. In cats with NFNFHD no adverse events were observed [18]; however, the dosages used in that study were the same as those used for dogs (0.4–0.6 mg/kg), which are lower than the dosages evaluated in the present study. It is important to evaluate the safety of oclacitinib in healthy cats to determine whether adverse events are likely to be due to the medication or other unrelated factors. To the author’s knowledge, this is the first report of the safety of higher dosages of oclacitinib in cats after twice-daily administration for 28 days in a placebo-controlled trial.

Oclacitinib has a more potent inhibitory activity against JAK-1, but also may inhibit JAK-2 [11], leading to a concern that it could cause bone marrow suppression. However, a study demonstrated that oclacitinib was much less potent against erythropoietin and granulocyte-macrophage colony-stimulating factor that are involved in hematopoiesis [11]. This could support the finding in the current trial that mean values of the red blood count, hemoglobin, and hematocrit were normal for both treated groups. Platelet mean values below the reference range in 1 mg/kg also occurred in the placebo group. Some cats in placebo and both treated groups had thrombocytopenia and platelet aggregates observed, which is a common problem in this specie. Furthermore, feline platelets are prone to clumping following blood collection, making it difficult to get accurate platelet counts. The variable size of feline platelets may be misidentified as erythrocytes and true thrombocytopenia occurs rarely in cats [25, 26].

Although there was a significant increase in neutrophils and monocytes mean counts in oclacitinib-treated group on day 21, values remained within the normal reference range and were accompanied by a non-significant increase in the leukocytes numbers. These increases may be due to excitement during blood sample collection, and monocytosis may occur any time that neutrophilia occurs [23]. At the subsequent sampling on day 28, mean counts presented no significant difference between the groups. In contrast, dogs that received oclacitinib presented a decrease in mean white blood cell, neutrophil, and monocyte counts [13, 14]. Mean eosinophil values were decreased in both groups treated with oclacitinib from day 3 until last day of treatment in comparison pretreatment values, but individual values were within the normal reference range and only significantly different from the placebo group only on day 14. This decrease in eosinophil values also was observed in dogs [13, 14]. A reduction in eosinophil counts was reported for cats treated with cyclosporine [7] and glucocorticoids [4].

Occasional serum chemistry values above the normal reference range observed in treated and placebo group were transient and not significantly different between treated and untreated groups, with the exception of fructosamine levels, which were significantly higher in both treated groups than in the placebo group on day 7, but were still within the normal reference range and remained stable during the trial. In addition, glucose levels remained normal throughout the study. It was concluded that the casts and crystals observed in urinalysis in treated and placebo groups have no clinical relevance as they can be found in normal urine samples [27]. A recent study reported that 4 of 14 cats treated with oclacitinib presented a mild increase in renal parameters [28], however, animals in the present study did not had any alteration in urea and creatinine values.

On the ultrasound examination, only one cat in 1 mg/kg treated group presented renal hyperechogenicity observed at the end of the study. This finding should not be considered an accurate indicator of renal disease due to its relatively high prevalence in small animals [29]. Furthermore, the cat in this study remained with normal levels of creatinine, urea, and UP/C. Cellularity observed in bladder ultrasonography in the animals in treated and placebo groups in this study has uncertain relevance since many normal cats may present with this finding without any clinical correlation [30].

Oclacitinib was judged to be well tolerated and easy to administer by cat owners in one study [18]. When efficacy of this drug in dogs was compared with prednisolone, both treatments were determined to be safe, rapid, and effective in controlling pruritus [31]. Results of the present study provide useful data regarding the safety of oclacitinib in cats, especially in cases that other medications, including glucocorticoids, are contraindicated. Future studies need to be performed to evaluate the effectiveness of both doses in pruritic cats and its long-term administration safety in this specie. We recognize that oclacitinib is not currently licensed for use in cats, and this paper may include references to products and formulations that are not available or licensed in the individual reader’s own country. Furthermore, some drugs mentioned may be licensed for human use and not for veterinary use.

## Conclusion

Oclacitinib was well tolerated by cats at 1 mg/kg and 2 mg/kg and appeared to be a safe medication for this specie to be treated twice daily for up to 28 days. More studies would be needed to demonstrate that oclacitinib maleate may be a suitable alternative to treat pruritic cats.

## Methods

### Animal population

The study was conducted at the Laboratory of Experimental Chemotherapy in Veterinary Parasitology of the Federal Rural of Rio de Janeiro University as a double-blinded placebo-controlled trial with a randomized complete block design. This study was submitted and approved by the Veterinary Institute Ethics Committee (CEUA/IV) (number 4534191017) and conducted in compliance with good animal management standards according to criteria defined by the ISFM - Feline-Friendly Handling Guidelines [32].

The study included 30 (15 male and 15 female) healthy, mixed-breed cats ranging in age from 2 to 3 years with weights ranging from 2.1 and 5.3 kg. The animals originated from the Laboratory of Experimental Chemotherapy in Veterinary Parasitology of the Federal Rural of Rio de Janeiro University available for animal experimentation. The cats were determined to be in good health on physical and clinical pathological evaluation before the initiation of the study. Only dewormed and vaccinated animals were selected to participate in the study; exclusion criteria included animals with clinically significant abnormalities and those requiring medication for any reason. Cats were housed in individual cages beginning on day − 7 (start of acclimation) through to the end of the study and were fed a habitual commercial dry feline diet every 12 h with fresh water provided ad libitum*.*

### Study design

Cats were randomly allocated according to sex and body weight (to avoid having to split tablets for dosing) to three treatment groups (placebo, oclacitinib maleate 1 mg/kg and oclacitinib maleate 2 mg/kg) with 10 animals in each group (5 males and 5 females). Cats in the placebo group received cornstarch tablets orally twice daily for 28 days. Cats in the 1 mg/kg and 2 mg/kg groups received oclacitinib tablets orally twice daily at 12 h- intervals for 28 days. All cats were weighed before treatment to calculate the appropriate dose. Cats were observed twice daily for general health, food consumption, urination, and signs of adverse effects, including vomiting or diarrhea throughout the entire study. Detailed clinical assessments of heart and lungs by auscultation, heart rate, respiratory rate, rectal temperature, mucous membranes, capillary refill time, hydration rate, abdomen, superficial lymph nodes, and oral cavity were performed by the same veterinarian on days − 2, 3, 7, 14, 21, 28, and 42. Animals also were weighed on these days and weights were recorded. Cats that could present serious adverse effect should have the treatment stopped.

Blood samples were collected on days of clinical assessments for hematology and serum chemistry analysis, including alanine aminotransferase, albumin, alkaline phosphatase, aspartate aminotransferase, cholesterol, creatinine, fructosamine, gamma glutamyl transpeptidase, glucose, bilirubins, total protein, triglycerides, and urea. Abdominal ultrasounds were performed to evaluate gastrointestinal, renal, and urinary tract status. Urine samples were collected by cystocentesis on days − 2, 14, 28, and 42 and submitted to the laboratory for routine urinalysis by accepted methods. All laboratory testing was performed on a blinded basis.

After the study the cats returned to the Laboratory of Experimental Chemotherapy in Veterinary Parasitology of the Federal Rural of Rio de Janeiro University.

### Statistical analysis

Following initial analysis of hematologic and serum chemistry results by the Shapiro-Wilks normality test, the Kruskal-Wallis test was used to test for differences between the groups followed by the Student-Newman-Keuls test at the 5% significance level. The confidence interval used for the statistical analysis was 95% (*p* < 0.05). Calculations were perfomed using the Biostat program version 5.3.

## Abbreviations

JAK: Janus kinase
NFNFHD: Non-flea, non-food hypersensitivity dermatitis
UP/C: Urine protein to creatinine ratio
